# Colonic Immune Stimulation by Targeted Oral Vaccine

**DOI:** 10.1371/journal.pone.0055143

**Published:** 2013-01-30

**Authors:** Mahesh Kathania, Mojgan Zadeh, Yaíma L. Lightfoot, Robert M. Roman, Bikash Sahay, Jeffrey R. Abbott, Mansour Mohamadzadeh

**Affiliations:** 1 Department of Infectious Diseases and Pathology, University of Florida, Gainesville, Florida, United States of America; 2 Division of Hepatology/Gastroenterology and Nutrition, University of Florida, Gainesville, Florida, United States of America; 3 Department of Medicine, Emerging Pathogens Institute, University of Florida, Gainesville, Florida, United States of America; The Ohio State University, United States of America

## Abstract

**Background:**

Currently, sufficient data exist to support the use of lactobacilli as candidates for the development of new oral targeted vaccines. To this end, we have previously shown that *Lactobacillus gasseri* expressing the protective antigen (PA) component of anthrax toxin genetically fused to a dendritic cell (DC)-binding peptide (DCpep) induced efficacious humoral and T cell-mediated immune responses against *Bacillus anthracis* Sterne challenge.

**Methodology/Principal Finding:**

In the present study, we investigated the effects of a dose dependent treatment of mice with *L. gasseri* expressing the PA-DCpep fusion protein on intestinal and systemic immune responses and confirmed its safety. Treatment of mice with different doses of *L. gasseri* expressing PA-DCpep stimulated colonic immune responses, resulting in the activation of innate immune cells, including dendritic cells, which induced robust Th1, Th17, CD4^+^Foxp3^+^ and CD8^+^Foxp3^+^ T cell immune responses. Notably, high doses of *L. gasseri* expressing PA-DCpep (10^12^ CFU) were not toxic to the mice. Treatment of mice with *L. gasseri* expressing PA-DCpep triggered phenotypic maturation and the release of proinflammatory cytokines by dendritic cells and macrophages. Moreover, treatment of mice with *L. gasseri* expressing PA-DCpep enhanced antibody immune responses, including IgA, IgG_1_, IgG_2b_, IgG_2c_ and IgG_3_. *L. gasseri* expressing PA-DCpep also increased the gene expression of numerous pattern recognition receptors, including Toll-like receptors, C-type lectin receptors and NOD-like receptors.

**Conclusion/Significance:**

These findings suggest that *L. gasseri* expressing PA-DCpep has substantial immunopotentiating properties, as it can induce humoral and T cell-mediated immune responses upon oral administration and may be used as a safe oral vaccine against anthrax challenge.

## Introduction

Mucosal surfaces are the principal sites of interaction between a microorganism and its host and, as such, represent the major route of entry for microbial pathogens [1]. In recent years, numerous reports of successful vaccination with mucosal vector vaccines have been published. The mucosal immune system functions to protect mucous membranes from invading infectious agents by regulating immune responses through selective, immune effector cascades, all of which are meant to protect the body from pathogen challenge [2]. Live bacteria and viruses are known to be more immunogenic than inactive vectors and thus, represent superior candidates to induce both mucosal and systemic immune responses against pathogens. The development of bacteria as live vaccine vehicles has focused primarily on the use of attenuated strains of pathogenic bacteria, including *Salmonella, Bordetella*, and *Listeria* spp. [3]–[5]. The pathogenic properties related to these bacteria render them attractive candidates to enhance immunogenicity; however, the potential toxicity and possibility of reversion of these attenuated strains to virulence is a significant safety concern. The strong immunogenicity of these vaccines also makes them less suitable for use in immunocompromised or susceptible individuals.

Consumption of fermented products, especially yogurt, has been recognized for centuries to have a positive effect on gastrointestinal health. These effects are now largely attributed to *Lactobacillus* species that are generally regarded as safe (GRAS) for human consumption. Lactic acid bacteria (LAB) comprise a group of Gram-positive bacteria that include species of *Lactobacillus, Lactococcus, Leuconostoc, Pediococcus*, and *Streptococcus* [6]. The ability of LAB to survive gastric transit and to assume a close physical association with the intestinal epithelium, in addition to their immunomodulatory properties and safe consumption in large quantities, make lactobacilli attractive candidates for the development of live vaccine vectors targeting immunogens to the intestinal mucosa [7]. Therefore, recent advances in biotechnology and in the understanding of intestinal immunity and microbial-host cell interactions have made it possible to design new mucosal delivery systems.

Vaccinations aimed at the mucosal immune system are intended to promote a robust systemic memory immune response that provides protection against repeat exposure to the targeted pathogen without causing tissue damage or excessive inflammation. Initiation and propagation of proinflammatory immune responses to infectious agents occurs through the activation of antigen-presenting cells (APCs) via innate receptors that bind conserved molecular patterns present on different groups of pathogens, or pathogen-associated molecular patterns. Toll-like receptors (TLRs), C-type lectin receptors (CLRs) and NOD-like receptors (NLRs) on these specialized phagocytic cells, which include DCs, recognize invading pathogens and trigger proinflammatory cytokines. Activation of DCs not only results in rapid proinflammatory cytokine secretion, but also induces the commitment of T cell subsets and subsequent B cell responses. Interestingly, specific *Lactobacillus* species have been shown to activate DCs, induce regulated inflammatory responses against infection, control the balance between Th1 and Th2 responses, and enhance IgA production [8], [9], further promoting their usefulness as live vaccine vectors.

Previously, we reported that recombinant *Lactobacillus acidophilus* or *Lactobacillus gasseri* expressing *Bacillus anthracis* protective antigen (PA) via specific DC-targeting peptides derived from a phage display peptide library elicited efficacious protective immunity against *B. anthracis* Sterne challenge [10], [11]. In the present study we evaluate the safety and immunogenicity of *L. gasseri* PA-DCpep and demonstrate that this candidate vaccine activates intestinal and systemic immunity.

## Materials and Methods

### Mice and Ethics Statement

C57BL/6 mice between 6 and 8 weeks of age were purchased from The Jackson Laboratories and were housed in the animal facility at the College of Veterinary Medicine, University of Florida. Procedures involving mice were in accordance with the Animal Welfare Act and the Public Health Service Policy on Humane Care and were approved by the Institutional Animal Care and Use Committee (IACUC) at the University of Florida.

### Bacterial Strains

*L. gasseri* PA-DCpep was inoculated at 1% and propagated in de Man, Rogosa, and Sharpe broth (Difco) at 37°C for 15 h supplemented with erythromycin (5 µg/mL). Subsequently, 1 mL of each culture was transferred to 10 mL of fresh de Man, Rogosa, and Sharpe broth and incubated at 37°C for 18 h supplemented with erythromycin (5 µg/mL). In preparation for oral treatment, the concentration of the *L. gasseri* strain was adjusted based on OD600 readings that had been previously correlated with CFU numbers [11]. *L. gasseri* expressing PA-DCpep was centrifuged, pelleted, and 10^7^, 10^9^ and 10^12^ CFU bacteria were re-suspended in 100 µL of PBS solution prior to oral gavage of the mice.

### Histopathology

C57BL/6 mice orally gavaged with increasing doses of *L. gasseri* expressing PA-DCpep (10^7^, 10^9^ or 10^12^ CFU) or PBS were sacrificed and their colons, livers, kidneys, spleens and ceca were surgically excised after days 1, 3, 7, and 14. The organs were fixed in formalin, embedded in paraffin, and tissue sections (4 µm) were stained with hematoxylin and eosin (H&E) and blindly scored, as described previously [12].

### Flow Cytometry

Single-cell suspensions of mesenteric lymph nodes (MLNs) from groups of mice that were treated with PBS or increasing doses of *L. gasseri* expressing PA-DCpep were filtered (70 µm) prior to staining with the specified surface markers and intracellular molecules. Lamina propria (LP) cells were also isolated from colons using collagenase treatment [13]. In brief, colons were flushed to wash off fecal content and opened longitudinally. Colons were then cut into 0.5 cm pieces, transferred to flasks and shaken for 25 min at 37°C in HBSS containing 5 mM EDTA and HEPES (10 mM) supplemented with 10% FBS. Cell suspensions were passed through a cell strainer and the remaining colonic tissue was washed with cold PBS, minced, transferred to conical flasks, and shaken for 25 min at 37°C in DMEM containing VII collagenase (1 mg/ml), HEPES (10 mM) CaCl_2_ (0.1M), and supplemented with 5% FBS. Cell suspensions were collected and passed through a strainer prior to staining and analysis; the resulting cell suspensions contained lamina propria lymphocytes (LPLs), our cells of interest. To exclude dead cells, a LIVE/DEAD Violet Dead Cell Stain kit was used (Invitrogen, Carlsbad, CA). Cells were washed and then incubated with Fc block (BD Bioscience, San Jose, CA). Subsequently, cells were stained with combinations of the following antibodies: CD11c PerCP-Cy5.5 (N418), CD40 PE (IC-10), FoxP3 PE (FJK-16A), IL-17 FITC (eBio17b7), CD86 APC (GL-1), RORγT APC (AFKJS-9; eBioscience, San Diego, CA), CD3 APC-Cy7 (145-2c11), CD45 APC-Cy7 (30-F11), CD4 PB (GK1.5), CD45 PB (30-F11), IL-10 FITC (JES5-16E3), CD8 PerCP-Cy5.5 (53-6.7), IFNγ PE-CY7 (XMG1.2), F4/80 PE-CY7 (BM8), B7-H1 PE-CY7 (10F.9G2), CD80 PB (16-10A1), IL-10 APC (JES5-16E3), IL-12 PE (C15.6), IL-22 APC (poly5164), CD11b APC-Cy7 (M1/70; Biolegend, San Diego, CA),TGFβ APC (R&D systems, Minneapolis, MN). Data were acquired with a FACSCanto II (BD) and analyzed by using FlowJo software (Tree Star).

### Sera Analyses

Cytokines and immunoglobulins systemically released into the peripheral blood of mice that were orally fed with different doses of *L. gasseri* expressing PA-DCpep or PBS were measured. Serum was collected from control and gavaged mice after days 1, 3, 7, and 14. Serum cytokines were measured using the mouse Th1/Th2/Th17 CBA kit from BD. The quantification of murine IgG_1_, IgG_2b_, IgG_2c_, IgG_3_, and IgA from sera was performed by using Ready-SET-Go!**®** Kits from eBioscience. Liver enzyme activity from serum was assessed using Aspartate Aminotransferase activity assay kit and Alanine Aminotransferase assay kit (BioVision).

### Real Time PCR

RNA was isolated from the mid section of the colons of control and *L. gasseri* expressing PA-DCpep-gavaged mice after days 1, 3, and 7 with Aurum**™** Total RNA Kit (Bio-Rad) according to the manufacturer’s instructions. RNA was reverse transcribed using iScript**™** select cDNA Synthesis kit (Bio-Rad) and cDNA was used for quantitative PCR by SYBR Green gene expression assay on Bio-Rad CFX96**™** Real time system. Primer sequences are listed in supplementary data (**Table. S1**). Samples were run in duplicate for each experimental condition and mean values were used to calculate statistics. GenePattern algorithm was used to generate heat maps and R software was used for Hierarchical matrix cluster analyses.

### Confocal Imaging

Colons of mice treated with increasing doses of *L. gasseri* expressing PA-DCpep or PBS alone were fillet-opened, rolled, and snap-frozen at −80°C in optimal cutting temperature (OCT) compound. Sections (5 µm) were cut, fixed in ice-cold methanol (−20°C) for 15 min, and blocked with Background Buster (Innovax™). Subsequently, sections were incubated overnight at 4°C with purified hamster anti-mouse CD11c (BD Biosciences) and purified CD11b rabbit anti-mouse (Abcam), CD4 mouse anti-mouse (Abcam) or B220 Rat anti-mouse (Abcam) and either rat anti-mouse IL-10 (BioLegend), goat anti-mouse IgA (Invitrogen), rat anti-mouse RORγT (Abcam), rat anti-mouse TNFα or rat anti-mouse IL-12. Sections were then washed three times with wash buffer (DAKO™), and incubated with anti-hamster Alexa-Fluor 647, anti-rabbit Alexa-Flour 555 and anti-rat Alexa-Fluor 488 (Invitrogen) for 1 h. Again, sections were then washed three times with wash buffer (DAKO™), incubated with DAPI (Invitrogen) for 10 min, washed twice with PBS, and mounted with antifade mounting medium. Images were acquired using LSM710 confocal microscope (Zeiss) using the Zen 2011 software.

### Statistical Analysis

All data are represented as mean ± SEM. Statistical analyses were performed using Student’s *t*- tests. *P<0.05 and **P<0.01 are considered statistically significant.

## Results

### *L. gasseri* Expressing PA-DCpep is Safe at High Doses

**Figure 1:**
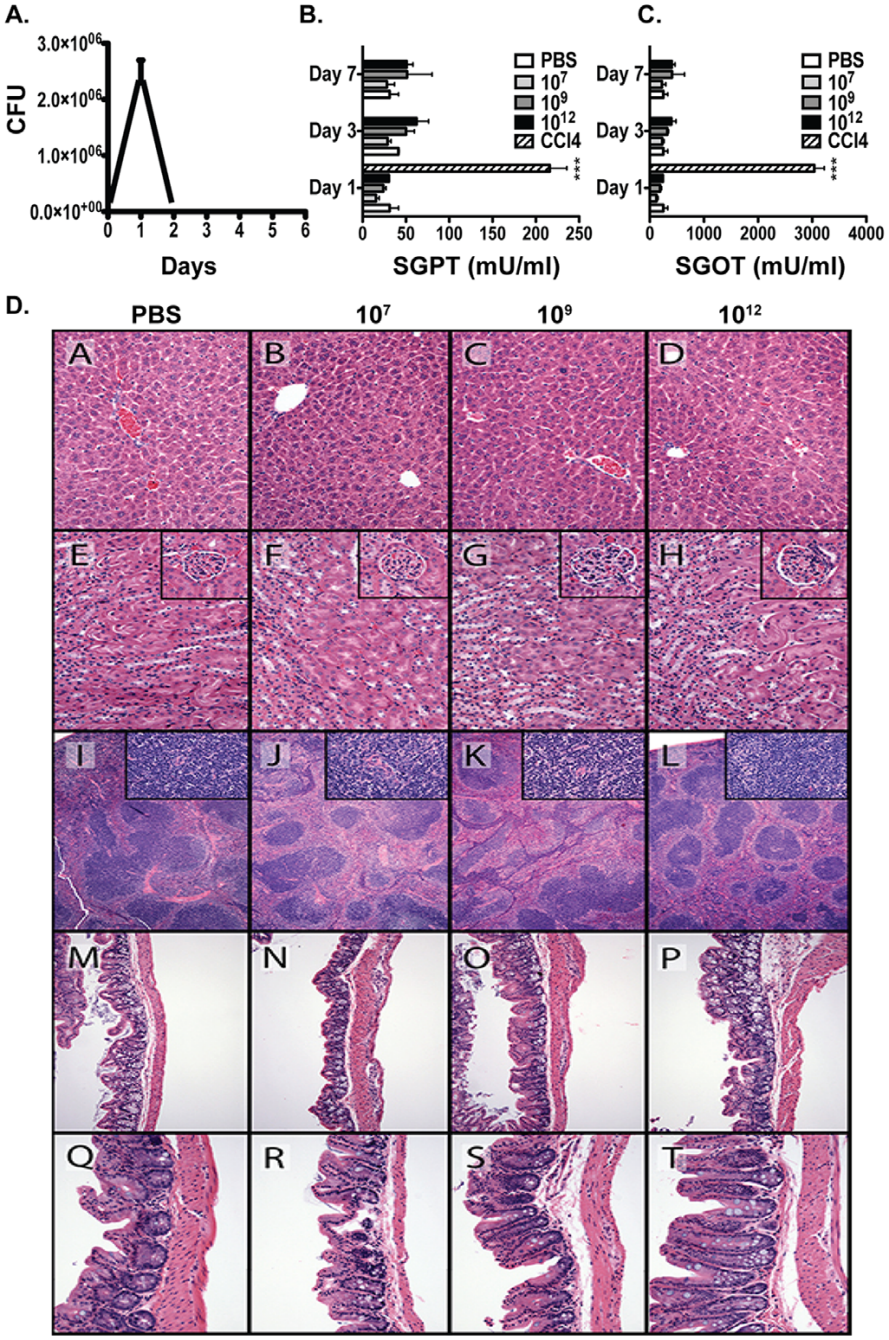
*L. gasseri* expressing PA-DCpep is safe for mice at high doses. C57BL/6 mice were orally gavaged with 10^9^ CFU of *L. gasseri* expressing PA-DCpep once and the CFU per gram of feces was determined (A) in MRS plates with erythromycin (5 µg/mL). C57BL/6 mice were orally gavaged with increasing doses of *L. gasseri* expressing PA-DCpep (10^7^, 10^9^ and 10^12^ CFU) or PBS, serum was collected after days 1, 3 and 7, and the enzyme activities of ALT/SGPT (B) and AST/SGOT (C) were analyzed by ELISA. CCl_4_ was used as a positive control for toxicity (B & C). Tissues from C57BL/6 mice orally gavaged with increasing doses of *L. gasseri* expressing PA-DCpep (10^7^, 10^9^ and 10^12^ CFU) or PBS were collected and sections stained with H&E at days 1, 3 and 7 of treatment. (D) Photomicrographs of H&E sections of liver A–D, kidney E–H, spleen I–L, colon M–P, and cecum Q–T. Insets of a representative glomerulus and surrounding proximal tubules are included in each of the kidney photomicrographs. Insets of high magnification of the lymphoid tissue are included in each of the spleen photomicrographs. For each of the organs, the PBS control is pictured in the left column A, E, I, M and Q, respectively. The three photomicrographs on the right are from increasing concentrations of bacteria from 10^7^, 10^9^ and 10^12^ from left to right.

In order to better understand immune activation and potential toxicities by *L. gasseri* expressing PA-DCpep, colonization of the gut by this bacterium in C57BL/6 mice was evaluated. The persistence of erythromycin-resistant *L. gasseri* expressing PA-DCpep in the colon was determined in C57BL/6 mice orally treated once with 1 × 10^9^ CFU/mouse. Fecal pellets were collected the day before treatment and every day for up to 8 days after bacterial ingestion. Data demonstrate that mice cleared *L. gasseri* expressing PA-DCpep after 2 days (**Fig. 1A**). Next, we evaluated potential hepatotoxicity by measuring the serum levels of the liver enzymes aspartate aminotransferase/serum glutamic oxaloacetic transaminase (AST/SGOT) and alanine aminotransferase/serum glutamic pyruvic transaminase (ALT/SGPT), which are increased with hepatocyte leakage or injury. The hepatotoxin, carbon tetrachloride (CCl_4_), was used as a positive control. Mice were treated orally with different doses of *L. gasseri* expressing PA-DCpep (10^7^, 10^9^ and 10^12^ CFU/mice) and sera collected after days 1, 3, 7, and 14 of gavage. **Fig. 1B, C** shows that, as measured by serum hepatic enzyme activity, there is no hepatotoxicity seen even with high doses of *L. gasseri* expressing PA-DCpep, while CCl_4_ treatment results in very high activities of AST/SGOT and ALT/SGPT, indicating severe hepatotoxicity. Histopathology of liver, kidney, spleen, colon, and cecum at days 1, 3, and 7-post administration of bacteria was evaluated at three different concentrations of bacteria (10^7^, 10^9^, and 10^12^) and the negative control, PBS. The only histopathologic lesions that differed from the PBS control were multifocal, minimal to mild lymphocytolysis in the lymphoid tissues of the spleen at all concentrations of bacteria on days 3 and 7-post inoculation. All other organs, regardless of day or bacterial concentration, were indistinguishable from the PBS control (**Fig. 1D**).

### Activation of DCs and Macrophages by *L. gasseri* Expressing PA-DCpep

**Figure 2:**
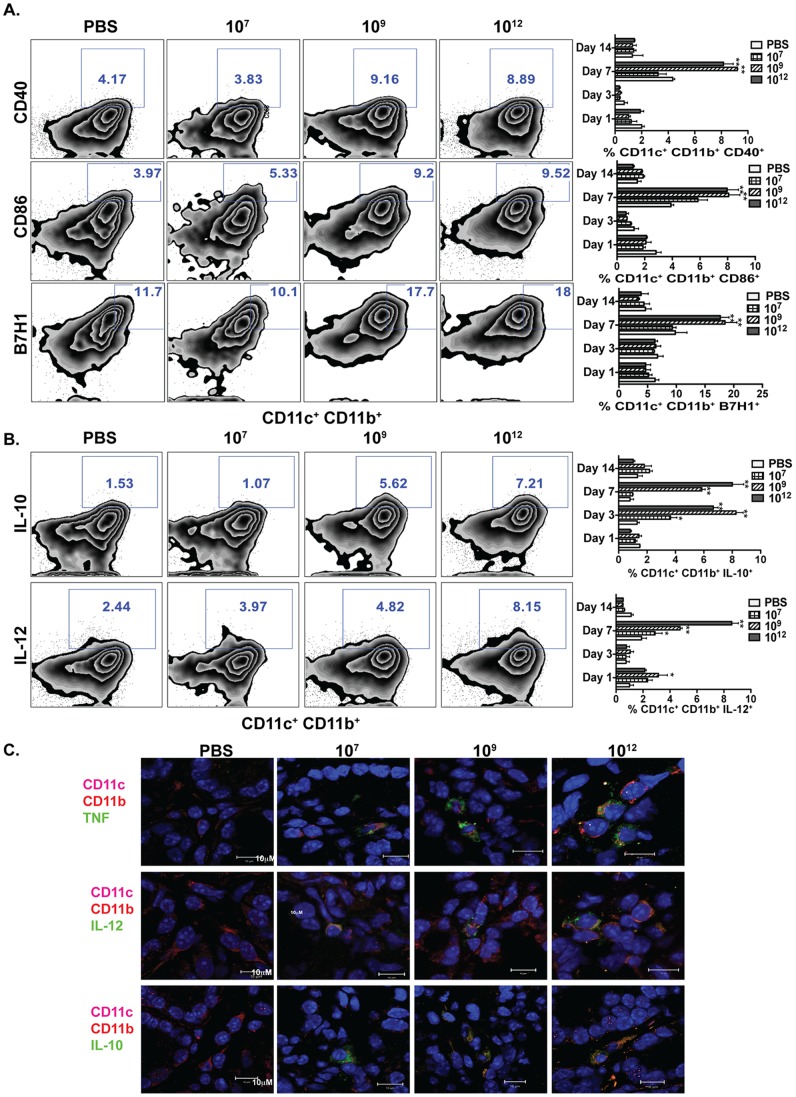
Activation of colonic DCs by *L. gasseri* expressing PA-DCpep. Groups of C57BL/6 mice (n = 3) were fed with different doses (10^7^, 10^9^ and 10^12^ CFU) of *L. gasseri* expressing PA-DCpep and sacrificed on days 1, 3, 7 and 14 post-inoculation. The activation of colonic DCs was evaluated by the surface expression of CD40, CD86 and B7H1, and analyzed by flow cytometry (A). Lamina propria lymphocytes (LPLs) were also stained with antibodies against CD11c, CD11b, IL-10 and IL-12, and analyzed by flow cytometry (B). Data are representative of two independent experiments. Error bars represent ±SEM. *P<0.05 and **P<0.01 compared with PBS. (C) C57BL/6 mice were orally gavaged with increasing doses of *L. gasseri* expressing PA-DCpep (10^7^, 10^9^ and 10^12^ CFU) or PBS and colonic sections were stained with CD11c (magenta), CD11b (red) and either TNFα, IL-12 or IL-10 (green) antibodies, and visualized using confocal microscopy.

Activation of immune cells was evident in C57BL/6 mice orally treated once with different doses of *L. gasseri* expressing PA-DCpep (10^7^, 10^9^, or 10^12^ CFU/mouse). Cells were isolated from the lamina propria using collagenase after days 1, 3, 7, and 14 of gavage. Cells were stained and analyzed by flow cytometry for evidence of activation of colonic DCs, macrophages, and T cells (**Fig. S1A**). To achieve full T cell activation, a second signal from co-stimulatory/regulatory molecules on APCs is required and thus, the expression of these molecules, including CD40, CD86, and B7-H1, was analyzed on DCs. We did not observe sustained activation of DCs in mice treated with *L. gasseri* alone past day 1 (Data Not Shown). However, a dose dependent increase in the surface expression of CD11c^+^CD11b^+^CD40^+^, CD86^+^ and B7-H1^+^ DCs was observed in *L. gasseri* expressing PA-DCpep-treated mice when compared to PBS-treated control mice (**Fig. 2A**). Activated DCs produced elevated levels of IL-10 and IL-12, as detected in colonic tissues by flow cytometry (**Fig. 2B**). Increased levels of IL-10, IL-12, and TNFα were also documented in CD11c^+^CD11b^+^ DCs by confocal imaging (**Fig. 2C**). Similarly, increased levels of IL-10 and IL-12 were measured in colonic macrophages from mice that were treated with *L. gasseri* expressing PA-DCpep (**Fig. S1B**).

### Activation of Th1, Th17, and Treg Cells by *L. gasseri* Expressing PA-DCpep

**Figure 3:**
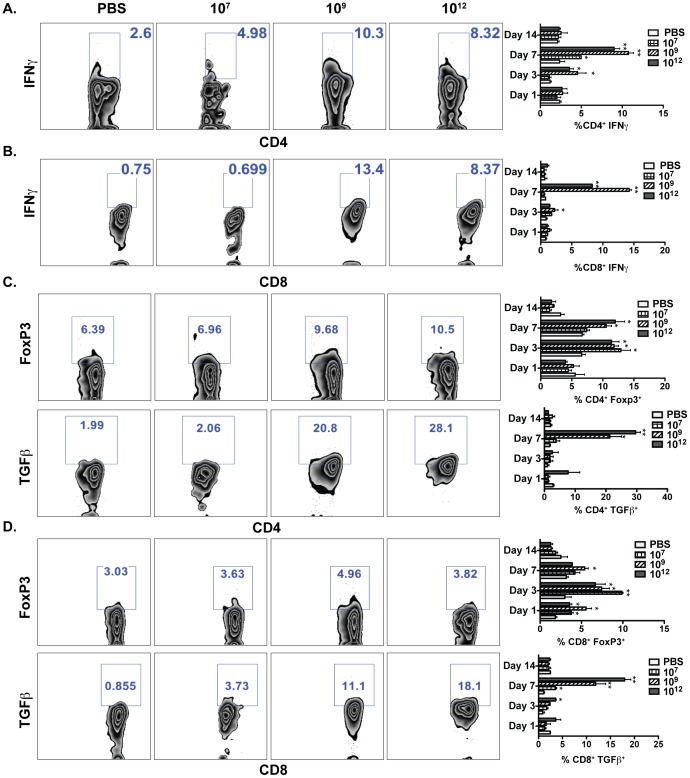
*L. gasseri* expressing PA-DCpep enhances the induction of regulatory T cells. C57BL/6 mice were orally gavaged with increasing doses of *L. gasseri* expressing PA-DCpep (10^7^, 10^9^ and 10^12^ CFU) or PBS; LPLs were harvested by collagenase digestion after days 1, 3, 7 and 14, stained with antibodies against CD4, CD8, IFNγ (A&B), TGFβ and FoxP3 (C&D), and analyzed by flow cytometry. Data are representative of two independent experiments. Error bars represent ±SEM. *P<0.05 and **P<0.01 compared with PBS.

To expand upon our cell activation findings, we studied the ability of *L. gasseri* expressing PA-DCpep treatment to induce the stimulation of CD4^+^ and CD8^+^ T cells. As seen in **Fig. 3A, B**, there was a marked increase in the production of IFNγ in both colonic CD4^+^ and CD8^+^ T cells of the mice that were treated with *L. gasseri* expressing PA-DCpep. In order to determine whether IFNγ production is negatively or positively regulated, we evaluated the activation of the T lymphocyte subsets, Tregs and Th17 cells, respectively. Colonic T cells were isolated after days 1, 3, 7, and 14 of treatment with *L. gasseri* expressing PA-DCpep, and cells were stained for FoxP3 and TGFβ. Our results clearly show that there is a significant increase in the production of TGFβ by both CD4^+^Foxp3^+^ and CD8^+^Foxp3^+^ T cells, indicating that there was an increase in Tregs after treatment with *L. gasseri* expressing PA-DCpep (**Fig. 3C, D**).

**Figure 4:**
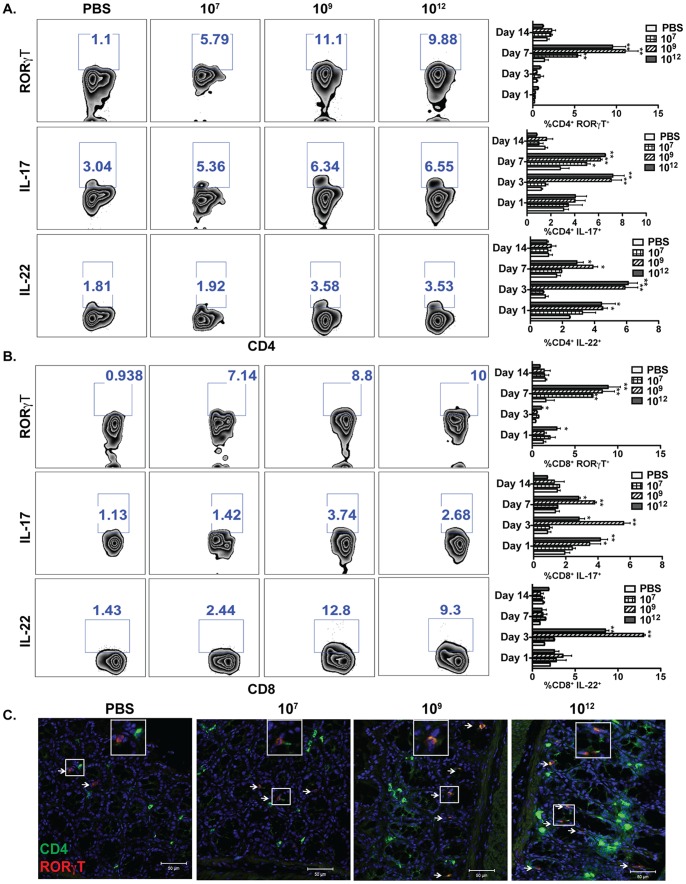
*L. gasseri* expressing PA-DCpep promotes Th17 responses. C57BL/6 mice were orally gavaged with increasing doses of *L. gasseri* expressing PA-DCpep (10^7^, 10^9^ and 10^12^ CFU) or PBS; LPLs were harvested by collagenase digestion after days 1, 3, 7 and 14, and stained with antibodies against CD4, CD8, RORγT, IL-17 and IL-22 (A&B) before analysis by flow cytometry. Data are representative of two independent experiments. Error bars represent ±SEM. *P<0.05 and **P<0.01 compared with PBS. (C) C57BL/6 mice were orally gavaged with increasing doses of *L. gasseri* expressing PA-DCpep (10^7^, 10^9^ and 10^12^ CFU) or PBS; colonic sections were stained with RORγT (red) and CD4 (green) antibodies, and visualized using confocal microscopy.

Subsequently, activation of the Th17 response was also studied in mice that were treated with *L. gasseri* expressing PA-DCpep. There was a significant increase in Th17 cells upon treatment with *L. gasseri* expressing the fusion protein, as seen by elevated expression of RORγT and increased production of IL-17 and IL-22 in CD4^+^ and CD8^+^ cells (**Fig. 4A, B**). Confocal imaging also showed that treatment of mice with *L. gasseri* expressing PA-DCpep increased the number of RORγT-expressing CD4^+^ T cells (**Fig. 4C**). Tregs play a key role in modulating the immune system. In this respect, the activation of Tregs indicates that the regulation of colonic Th1 and Th17 immune responses by *L. gasseri* expressing PA-DCpep may be highly controlled by colonic Tregs.

### *L. gasseri* Expressing PA-DCpep Induces Systemic Immune Activation

In order to evaluate the effects of *L. gasseri* expressing PA-DCpep on the immune responses in mesenteric lymph nodes (MLNs) of mice that were treated with different doses of *L. gasseri* expressing PA-DCpep, immune cells were stained and analyzed by flow cytometry. Once again, increased frequencies of CD11c^+^CD11b^+^CD80^+^ and CD11c^+^CD11b^+^B7-H1^+^ cells were seen in the MLNs of mice treated with *L. gasseri* expressing PA-DCpep (**Fig. S2**). The production of cytokines, including IL-10, IL-12 and TNFα was also induced in both DCs and macrophages (**Fig. S3)**.

T cell activation stimulated by *L. gasseri* expressing PA-DCpep was also analyzed in MLNs. As in the colon, mice treated with *L. gasseri* expressing PA-DCpep showed increased production of IL-10 and TGFβ, as well as increased expression of FoxP3 in CD4^+^ and CD8^+^ T cells derived from MLNs when compared to PBS treated mice (**Fig. S4**). There was also an increase in CD4^+^ and CD8^+^ Th17 cells, as measured by higher expression of RORγT and enhanced levels of IL-17, IFNγ and IL-22 in *L. gasseri* expressing PA-DCpep-treated mice (**Fig. S5**). Th1 cells were also activated, as both CD4^+^ and CD8^+^ T cells showed enhanced IFNγ production with increasing doses of *L. gasseri* expressing PA-DCpep (**Fig. S5**).

### Modulation of Colonic Pattern Recognition Receptor Gene Expression by *L. gasseri* Expressing PA-DCpep

Innate immunity plays a key role in the development of protective adaptive immune responses during vaccination. Many adjuvants are used in vaccine development to activate innate immune cells, which in turn, release cytokines and chemokines for adequate communication between different cellular components of immunity. We have noted above the activation of DCs and macrophages upon treatment with the vaccine under study (**Figs. 2** **, S1B**). Activation of innate immune cells such as macrophages and DCs also activates the expression of various innate immune receptors on their cell surfaces, which further primes the immune system for additional immune challenges and contributes to the milieu of cytokine and chemokine mediators. To test whether *L. gasseri* expressing PA-DCpep elevates the expression of these innate immune receptors, we performed quantitative real-time PCR to identify the genes differentially expressed in the colons of mice gavaged with increasing doses of *L. gasseri* expressing PA-DCpep.

**Figure 5:**
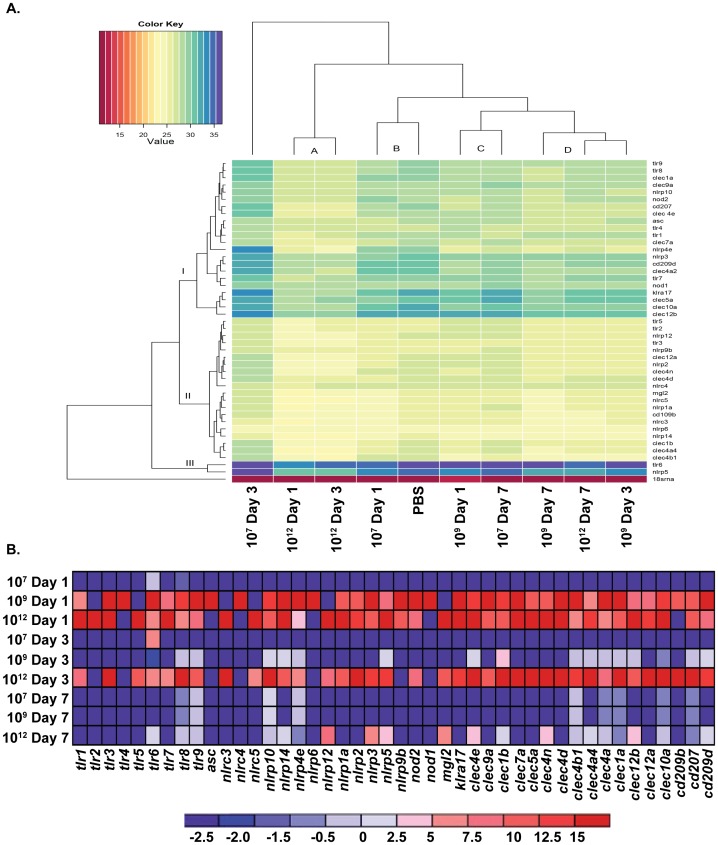
Activation of PRR-genes by *L. gasseri* expressing PA-DCpep. C57BL/6 mice were orally gavaged with increasing doses of *L. gasseri* expressing PA-DCpep (10^7^, 10^9^ and 10^12^ CFU), or PBS; RNA was isolated from colon tissue after days 1, 3 and 7 of treatment. Quantitative real-time PCR was performed to measure changes in gene expression. Hierarchical matrix cluster analysis (A) and comparative gene analysis presented as a heat map (B). Data are representative of two independent experiments with duplicates.

To understand the transcriptional regulation of these genes in the colon amongst the different treatment groups, we carried out a Hierarchical matrix cluster analysis with Euclidean distance. The consensus clustering of the differentially expressed genes unravels gene clusters with distinct expression time profiles (**Fig. 5A**). The threshold cycles of gene expression used for this analysis revealed four distinct clusters among the samples. Cluster A contains colon samples after days one and three-post gavage with 10^12^ *L. gasseri* expressing PA-DCpep. This group demonstrated the highest levels of gene expression among all samples. The mouse that had received 10^7^ bacteria one day prior to analysis clustered together with the mouse that received PBS, suggesting minimal changes in gene expression. Cluster C includes samples from the mice gavaged with 10^9^ and 10^7^ bacteria at days one and seven, respectively. The colon samples collected from the mice three and seven days post-10^9^ bacterial treatments, along with the colon samples isolated from the mice seven days post-10^12^ bacterial gavage, grouped in cluster D. This cluster analysis demonstrated that increasing doses of vaccine enhanced the expression of innate immune receptors and that the increased expression was maintained for a longer period of time. The transcripts were grouped into three distinct clades; clade II contains the genes highly expressed in the colon followed by clades I and III, which contain the genes moderately and least expressed in the colon samples tested, respectively.

The genes grouped in clade II contain several genes that regulate vital functions in gut immunity and adaptive immune responses. These genes, including *tlr2, tlr3, tlr5, clec12a, nlrp2 nlrc4, nlrp1a* and *nlrcp6*, enhance adjuvanticity via the activation of DCs and other innate immune cells. We also observed higher expression of negative regulatory genes such as, *nlrp12, nlrc4,* and *nlrc5,* which are critical for the counterbalance of the exacerbated inflammation due to hyperactivation of innate immune cells. Similarly, *nlrp6* and *nlrp10,* found to be elevated and depicted in the clade I, are responsible for providing regulatory signals for homeostasis and for providing signals to DCs resulting in the migration to lymph nodes to induce adaptive immune reactions. Moreover, we have observed the induction of Th17 cells and their capacity to release signature cytokines, IL-17 and IL-22. The activation of this T cell subset is reported to be dependent upon certain innate immune responses, in particular DCs expressing *tlr1, tlr2* and *clec4n*, which are elevated upon bacterial treatment [14], [15].

Furthermore, investigation of the pattern recognition receptor (PRR) genes in the colons of bacteria-treated mice revealed a significant activation in the expression of TLR, NLR, and CLR genes compared to PBS controls (**Fig. 5B**). The majority of the TLR (*tlr 1, 6, 7, 8, 9*), NLR (*nlrp3, 4e, 5, 10,12,14*) and CLR (*clec1a, 1b, 4a, 4a4, 4b, 4e, 10a, klra 17, cd209d, cd207*) genes were activated after day 1 of gavage at doses of 10^9^ and 10^12^ CFU; however, most of the gene activation was reduced in subsequent days of treatment. This change in gene expression suggests that colonic DCs and macrophages of mice gavaged with *L. gasseri* expressing PA-DCpep underwent activation. Such DC or macrophage activation occurs collectively via a complex cascade of PRRs *in vivo* that might be activated to mobilize immunity against pathogen challenge. These data demonstrate an efficacious vaccine inducing the activation of various PRRs, all of which act to orchestrate optimal immune responses against pathogen challenge.

### Activation of Humoral Immune Response and Cytokine Production

**Figure 6:**
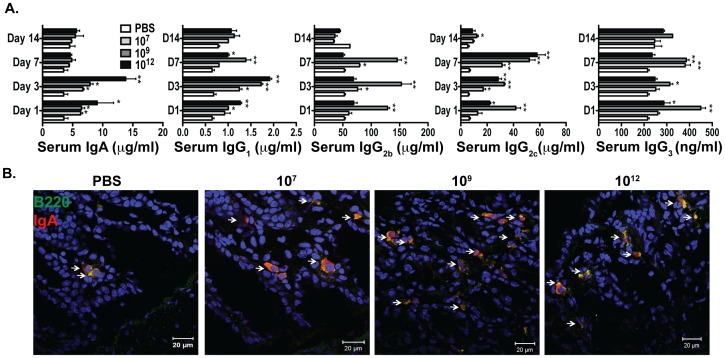
Humoral immune responses elicited by *L. gasseri* expressing PA-DCpep. C57BL/6 mice were orally gavaged with increasing doses of *L. gasseri* expressing PA-DCpep (10^7^, 10^9^ and 10^12^ CFU) or PBS; serum was isolated after days 1, 3, 7 and 14, and ELISAs were performed to measure the secretion of IgA, IgG_1_, IgG_2b_, IgG_2c_ and IgG_3_ (A). Data are representative of two independent experiments. Error bars represent ±SEM. *P<0.05 and **P<0.01 compared with PBS. (B) C57BL/6 mice were orally gavaged with increasing doses of *L. gasseri* expressing PA-DCpep (10^7^, 10^9^ and 10^12^ CFU) or PBS; colonic sections were stained with B220 (green) and IgA (red) antibodies, and visualized using confocal microscopy.

To assess the mucosal and systemic humoral immune responses induced by the oral administration of *L. gasseri* expressing PA-DCpep, the colons and sera of treated and untreated mice were tested for IgA and IgG subclasses by confocal imaging and ELISA, respectively. Mice treated with *L. gasseri* expressing PA-DCpep produced higher levels of IgA compared to PBS-treated mice (**Fig. 6A and B**). In addition, sera derived from mice gavaged with *L. gasseri* expressing PA-DCpep showed higher serum levels of IgG-subclasses, including IgG_1_, IgG_2b,_ IgG_2c_ and IgG_3_, after days 1, 3, and 7, with significant reductions thereafter (**Fig. 6A**). The secretion of cytokines was also measured in the sera, which showed increased production of IFNγ, TNFα, IL-17, IL-6, and IL-10 in mice treated with *L. gasseri* expressing PA-DCpep (**Fig. S6**).

## Discussion

To achieve potent vaccination against microbial challenge, various platforms have been tested; however, many of these vaccine strategies still need to be refined with the use of cellular and molecular approaches [16]. Currently, two licensed anthrax vaccines are available: AVP and AVA/Biothrax. The major drawbacks of these vaccines are that they induce only limited protection and require frequent vaccinations to provide protective immunity [17]. Moreover, there is significant variability in the protection conferred among different animal species [18]. Various research groups have tested intranasal vaccine delivery with different vaccine formulations [2], [19], [20]. Such work has correlated protection against virulent anthrax challenge with boosting mucosal immunity following intranasal and subcutaneous administration of recombinant PA plus formaldehyde-inactivated spores. Another potential strategy against anthrax consists of orally vaccinating with a live vector to induce protective immunity in both the lungs and intestines. Several vectors for PA-expression have been tested for the ability to elicit mucosal PA-specific immunity (i.e., live *B. anthracis* Sterne strain, *B. subtilis*, and *Salmonella*) [16]. Each of these vectors pose significant safety concerns related to their pathogenicity, such as possible adverse immune effects and the potential to revert to virulence [21]. Accordingly, the limitations of the anthrax vaccines available have encouraged research to improve vaccination formulations and the routes of vaccine administration.

The establishment of oral vaccines for protection against pathogens requires critical parameters, including excellent antigen delivery and presentation *in vivo*. Consequently, vaccines should be protected from enzymatic digestion and physical elimination that proves “non-productive”, as well as target professional DCs situated in the mucosal side of the host specifically. It is noteworthy that the vaccine delivery route is critical to establish efficacious mucosal vaccination. One of these delivery routes for mucosal vaccination is oral administration that, upon contact with DCs in gut-associated lymphoid tissue (GALT), provides protective immunity against invading pathogens [22]. Therefore, targeting anthrax PA to intestinal DCs would mobilize not only effective mucosal, but also systemic protection against pathogen challenge. Yet, the question of how to deliver a vaccine to intestinal DCs to initiate protective immunity without impairing their function remains.

Here, we offer a novel strategy for the development of such an oral vaccine that comprises a natural delivery vector using the specific probiotic, *L. gasseri*, which simultaneously serves as an adjuvant [9], and expresses the fusion of an antigen and a 12-mer peptide that specifically binds with high affinity to intestinal DCs [10], [23]. Our current work takes advantage of recent immunologic advances to develop a vaccine, which a) is designed to circumvent tolerance by oral vaccination with a mucosal vaccine; b) will be used in infectious disease states for protection against a deadly pathogen that threatens public health; and c) activates intestinal DCs that co-stimulate CD4^+^ T cell responses specifically to augment weak immune responses, resulting in robust T cell memory. To further improve our oral targeted vaccine comprised of anthrax PA and DCpep expressed by *L. gasseri* that induces protective immune responses against anthrax Sterne strain challenge, we addressed several questions, including the breadth of humoral and T cell immune responses, and more importantly, the safety of such an oral targeted vaccine *in vivo*.

We have recently demonstrated that treatment of mice with *L. acidophilus and L. gasseri* expressing PA-DCpep resulted in complete survival of animals challenged with *B. anthracis* Sterne [10], [11]. Hence, safety evaluation of the vaccine in mice gavaged with increasing doses of *L. gasseri* expressing PA-DCpep was performed. As seen in **Figure 1**, no significant toxic effects of high doses of the vaccine was observed when assessed by liver enzyme activity or H&E staining of different organs. Efficient vaccination against infectious agents depends upon specific antigen targeting to DCs. Vaccines containing a DC-binding peptide conjugated to an immunogenic vaccine subunit can facilitate the rapid capture of immunogenic antigens by DCs [10], which in turn, induce the activation and differentiation of different T cell subsets. Accordingly, we evaluated the cell-mediated immune responses generated by *L. gasseri* expressing PA-DCpep.

Our results demonstrate that *L. gasseri* expressing PA-DCpep optimally induces the activation innate immune cells, including DCs, by enhancing co-stimulatory/co-regulatory molecule expression, such as CD40, CD80, B7-H1, IL-12, TNFα, and IL-10 (**Fig. 2**). Such activation of innate cells (DCs) by a targeted vaccine is critical to subsequent activation and differentiation of T cells [24] in order to mobilize immunity against pathogens. In this regard, there is an increased level of IFNγ production by both CD4^+^ and CD8^+^ T cells (**Fig. 3** **A&B**). Our results support the previous findings that IFNγ-producing CD4^+^ T lymphocytes induce protective immunity to encapsulated *B. anthracis* [25]. IFNγ is a cytokine that is secreted by T lymphocytes and NK cells and leads to increased phagocytic activity which effectively kills pathogens, modulates chemotaxis, and upregulates antigen presentation to skew T cells toward a Th1 phenotype against pathogen challenge [26]. Importantly, we also observed significant recruitment of induced CD4^+^ and CD8^+^ Tregs, as well as Th17 cells upon oral treatment of mice with *L. gasseri* expressing PA-DCpep (**Fig. 3** **C&D,** **Fig. 4**). Tregs represent suppressive cell subsets, which act to regulate T cell responses and are thereby, thought to prevent pathology resulting from excessive immune responses [27]. On the other hand, it has been shown that protection against anthrax inhalation by an irradiated spore vaccine depends on cholera toxin adjuvant activity mediated by the induction of CD4^+^ Th17 cells [28]. This T cell subset is known to be important in mucosal adjuvant activity by improving epithelial barrier function, neutrophil recruitment, and enhanced antibody production and secretion [29].

To shed light on the humoral immune response, antibody production was also examined, as complete protection against pathogen challenge requires the contribution of a robust humoral immune response. The role of secretory IgA at mucosal surfaces and its role in the protection against *B. anthracis* has already been established [10]. Antigen-specific secretory IgA can exert its beneficial effects by interfering with the early events of infection through the inhibition of bacterial attachment and colonization, or by facilitating phagocytosis and subsequent antigen presentation by DCs and macrophages [30]. *L. gasseri* expressing PA-DCpep induced a significant amount of IgA compared to PBS-treated mice (**Fig. 6** **A&B**). It has been reported previously that anti-PA antibodies have anti-spore activity and thus might have a role in impeding the early stages of infection with *B. anthracis* [31]. Our work has shown that *L. gasseri* expressing PA-DCpep enhanced PA-specific antibody secretion and IgA levels, which might be important in inducing protective immunity against anthrax, as this subclass of immunoglobulin is increased in protected mice upon Sterne strain challenge [10]. Other immunoglobulins, which might be involved in immune protection, were also evaluated, and increased levels of serum IgG_1_, IgG_2b_, IgG_2c_ and IgG_3_ were seen in mice gavaged with *L. gasseri* expressing PA-DCpep (**Fig. 6A**). Antibody subclass switching to IgG_2b_, IgG_2c_ and IgG_3_ are induced by Th1 cytokines (IFNγ), whereas IgG_1_ is induced by Th2 cytokines. Studies are currently in progress to understand the role of B1 and B2 cell subsets in mice upon treatment with our targeted oral vaccine.

Furthermore, activation of TLRs has been correlated with the maturation and recruitment of DCs and macrophages [32]. Whereas activation of NLR genes leads to inflammasome activation resulting in the release of proinflammatory cytokines [33], CLR gene activation leads to more efficient processing and presentation of antigens on MHC-II [34]. Our results show that significant activation of all three classes of PRRs, including TLRs, NLRs and CLRs, may be required to achieve efficacious immunity against microbial infection (**Fig. 5**).

In light of these findings, it becomes clear that *L. gasseri* expressing PA-DCpep induces the activation and maturation of innate cells, thereby increasing the efficiency of antigen presentation, and also activates mucosal and systemic immunity via inflammatory/regulatory cytokines to ultimately establish functional immune responses against pathogen challenge. In conclusion, our recombinant *L. gasseri* expressing PA-DCpep may provide a highly efficient, cost effective, and safe targeted vaccine platform against *B. anthracis* that can be tested in higher animal models.

## Supporting Information

Figure S1 ***L. gasseri*** **expressing PA-DCpep induces cytokine secretion in colonic macrophages. (**A) Gating strategy of lamina propria cells for T cells and DCs. (B) C57BL/6 mice were orally gavaged with increasing doses of *L. gasseri* expressing PA-DCpep (10^7^, 10^9^ and 10^12^ CFU) or PBS; LPLs were harvested by collagenase treatment after days 1, 3, 7 and 14, and were stained with antibodies against CD11c, CD11b, F4/80, IL-10 and IL-12 before analysis by flow cytometry.
(TIF)

Figure S2 ***L. gasseri*** **expressing PA-DCpep activates DCs in MLN.** C57BL/6 mice were orally gavaged with increasing doses of *L. gasseri* expressing PA-DCpep (10^7^, 10^9^ and 10^12^ CFU) or PBS, and sacrificed on days 1, 3, 7 and 14 post-inoculation. The activation of DCs in MLNs was evaluated by surface expression of CD40, CD80 and B7H1, and analyzed by flow cytometry. Data are representative of two independent experiments. Error bars represent ±SEM. *P<0.05 and **P<0.01 compared with PBS.
(TIF)

Figure S3 ***L. gasseri*** **expressing PA-DCpep induces systemic immunity. (**A & B) C57BL/6 mice were orally gavaged with increasing doses of *L. gasseri* expressing PA-DCpep (10^7^, 10^9^ and 10^12^ CFU) or PBS; MLNs were harvested after days 1, 3, 7 and 14, stained with antibodies against CD11c, CD11b, F4/80, IL-10, IL-12 and TNFα, and analyzed by flow cytometry. Data are representative of two independent experiments. Error bars represent ±SEM. *P<0.05 and **P<0.01 compared with PBS.
(TIF)

Figure S4 ***L. gasseri*** **expressing PA-DCpep induce regulatory T cells in MLN. (**A & B) C57BL/6 mice were orally gavaged with increasing doses of *L. gasseri* expressing PA-DCpep (10^7^, 10^9^ and 10^12^ CFU) or PBS, and MLNs were harvested after days 1, 3, 7 and 14, stained with antibodies against CD4, CD8, FoxP3, TGFβ and IL-10, and analyzed by flow cytometry. Data are representative of two independent experiments. Error bars represent ±SEM. *P<0.05 and **P<0.01 compared with PBS.
(TIF)

Figure S5 ***L. gasseri*** **expressing PA-DCpep induce Th17 and Th1 cells in MLNs. (**A & B) C57BL/6 mice were orally gavaged with increasing doses of *L. gasseri* expressing PA-DCpep (10^7^, 10^9^ and 10^12^ CFU) or PBS; MLNs were harvested after days 1, 3, 7 and 14, and stained with antibodies against CD4, CD8, RORγT, IL-17, IL-22 and IFNγ, and analyzed by flow cytometry. Data are representative of two independent experiments. Error bars represent ±SEM. *P<0.05 and **P<0.01 compared with PBS.
(TIF)

Figure S6 **Augmentation of sera-cytokines by** ***L. gasseri*** **expressing PA-DCpep.** C57BL/6 mice were orally gavaged with increasing doses of *L. gasseri* expressing PA-DCpep (10^7^, 10^9^ and 10^12^ CFU) or PBS; serum was collected after days 1, 3 and 7. ELISAs were performed to measure the secretion of cytokines. Data are representative of two independent experiments.
(TIF)

Table S1 Primer Sequence used for qPCR.
(DOCX)
