# Supplementary figures and images for: Colonic Immune Stimulation by Targeted Oral Vaccine
Source: PLoS One. 2013 Jan 30;8(1):e55143. doi: 10.1371/journal.pone.0055143 (PMC3559436; doi:10.1371/journal.pone.0055143)

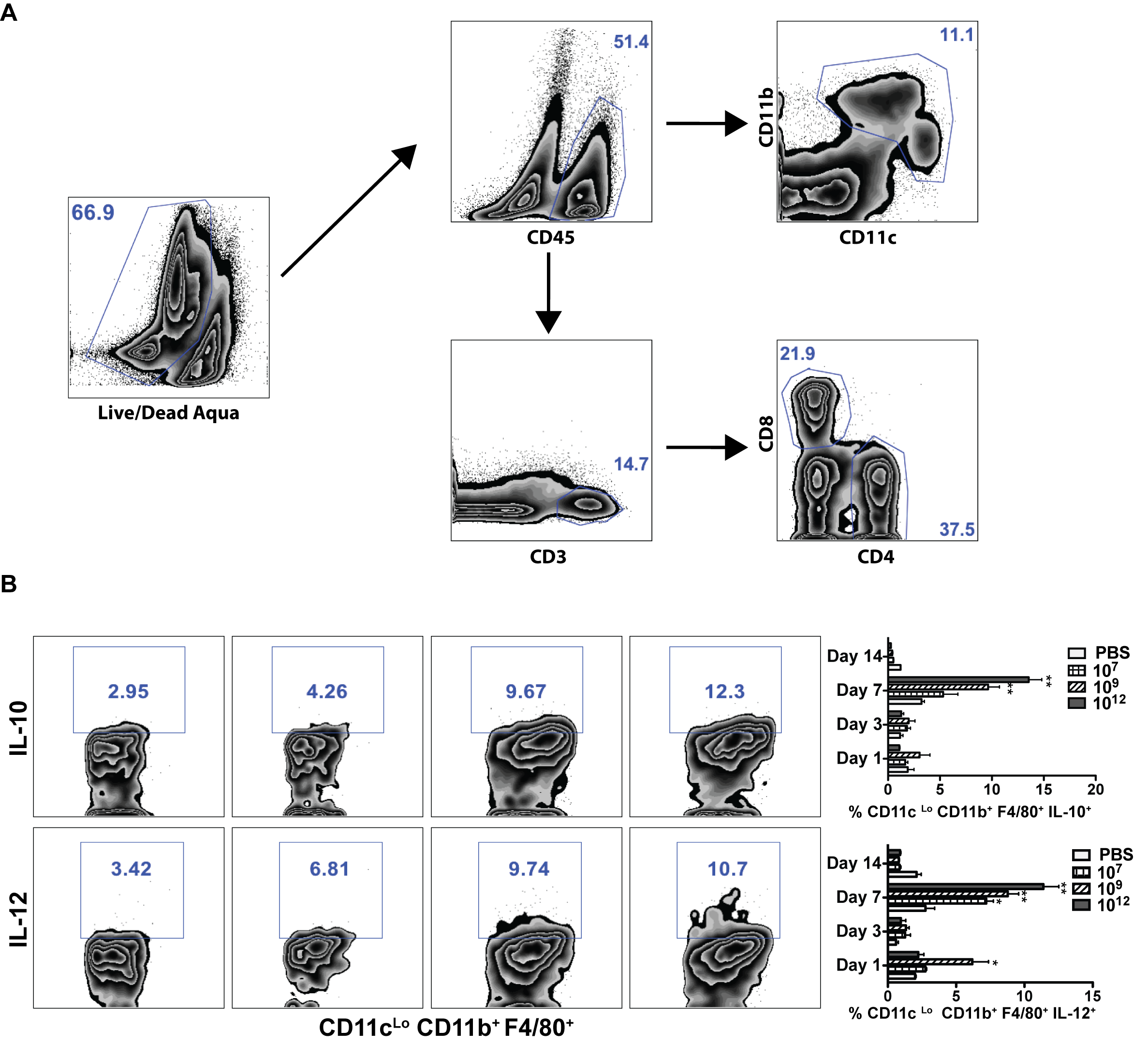

Supplement: Figure S1 — L. gasseri expressing PA-DCpep induces cytokine secretion in colonic macrophages. (A) Gating strategy of lamina propria cells for T cells and DCs. (B) C57BL/6 mice were orally gavaged with increasing doses of L. gasseri expressing PA-DCpep (107, 109 and 1012 CFU) or PBS; LPLs were harvested by collagenase treatment after days 1, 3, 7 and 14, and were stained with antibodies against CD11c, CD11b, F4/80, IL-10 and IL-12 before analysis by flow cytometry. (TIF) [file pone.0055143.s001.tif]

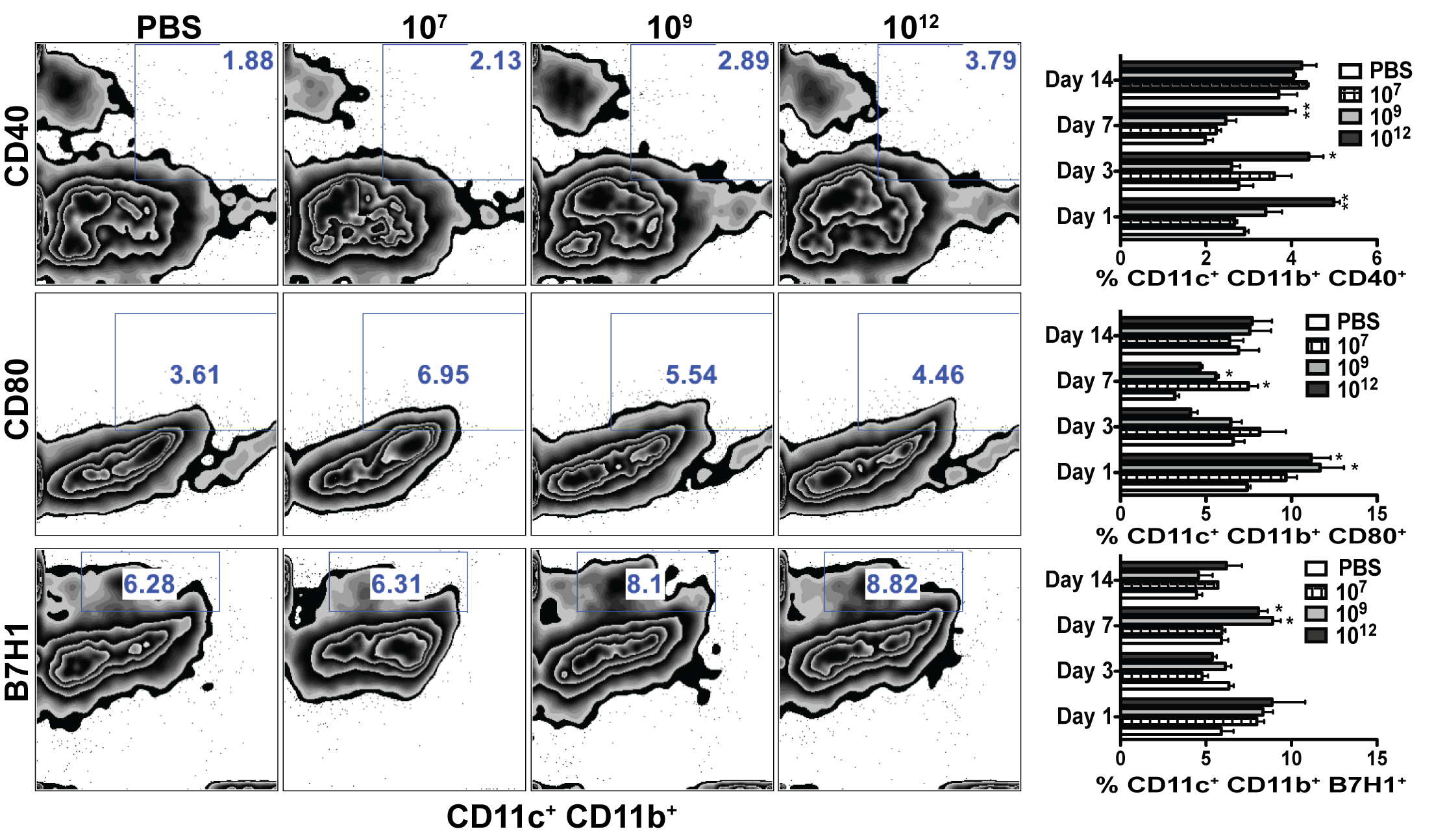

Supplement: Figure S2 — L. gasseri expressing PA-DCpep activates DCs in MLN. C57BL/6 mice were orally gavaged with increasing doses of L. gasseri expressing PA-DCpep (107, 109 and 1012 CFU) or PBS, and sacrificed on days 1, 3, 7 and 14 post-inoculation. The activation of DCs in MLNs was evaluated by surface expression of CD40, CD80 and B7H1, and analyzed by flow cytometry. Data are representative of two independent experiments. Error bars represent ±SEM. *P<0.05 and **P<0.01 compared with PBS. (TIF) [file pone.0055143.s002.tif]

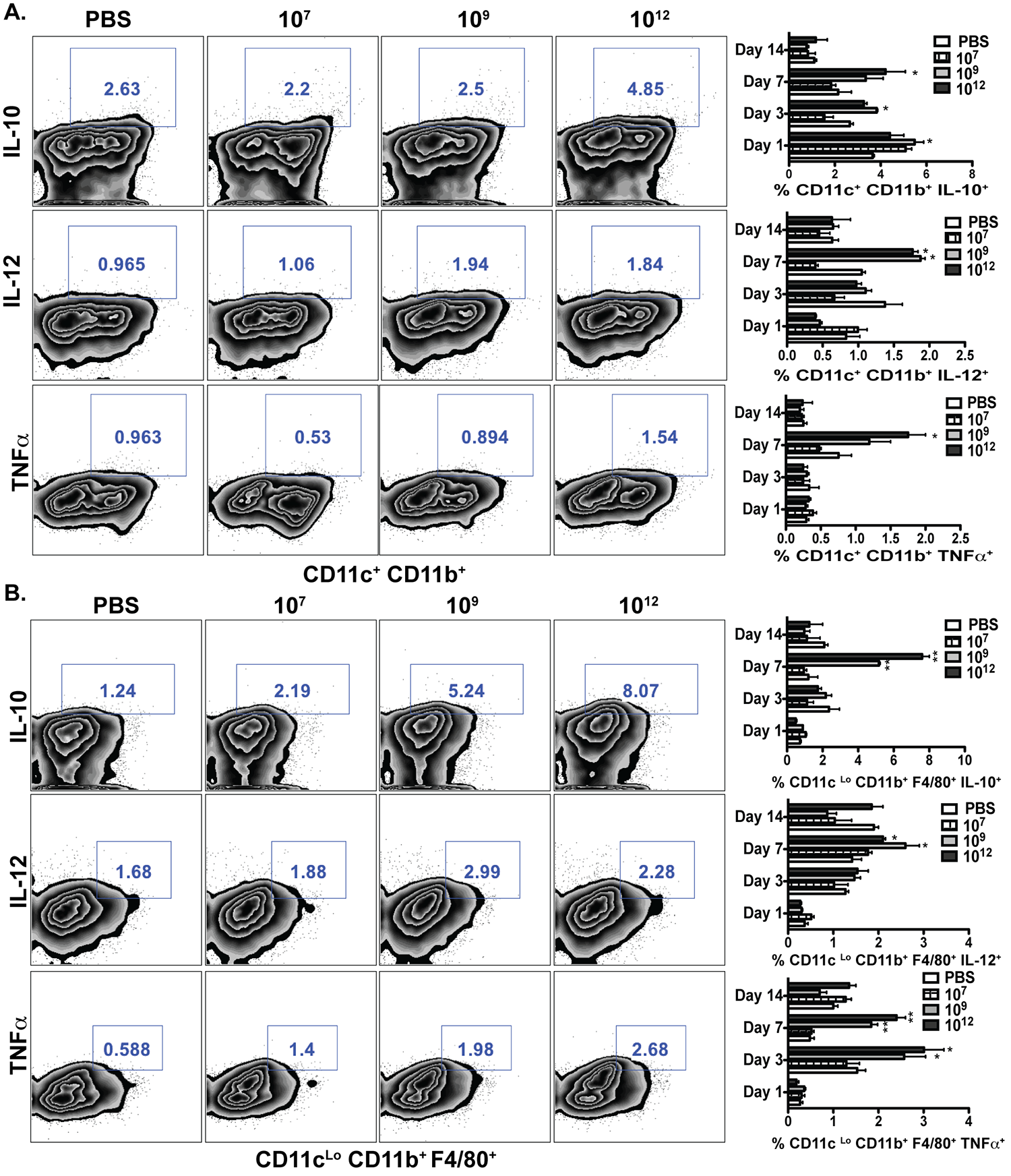

Supplement: Figure S3 — L. gasseri expressing PA-DCpep induces systemic immunity. (A & B) C57BL/6 mice were orally gavaged with increasing doses of L. gasseri expressing PA-DCpep (107, 109 and 1012 CFU) or PBS; MLNs were harvested after days 1, 3, 7 and 14, stained with antibodies against CD11c, CD11b, F4/80, IL-10, IL-12 and TNFα, and analyzed by flow cytometry. Data are representative of two independent experiments. Error bars represent ±SEM. *P<0.05 and **P<0.01 compared with PBS. (TIF) [file pone.0055143.s003.tif]

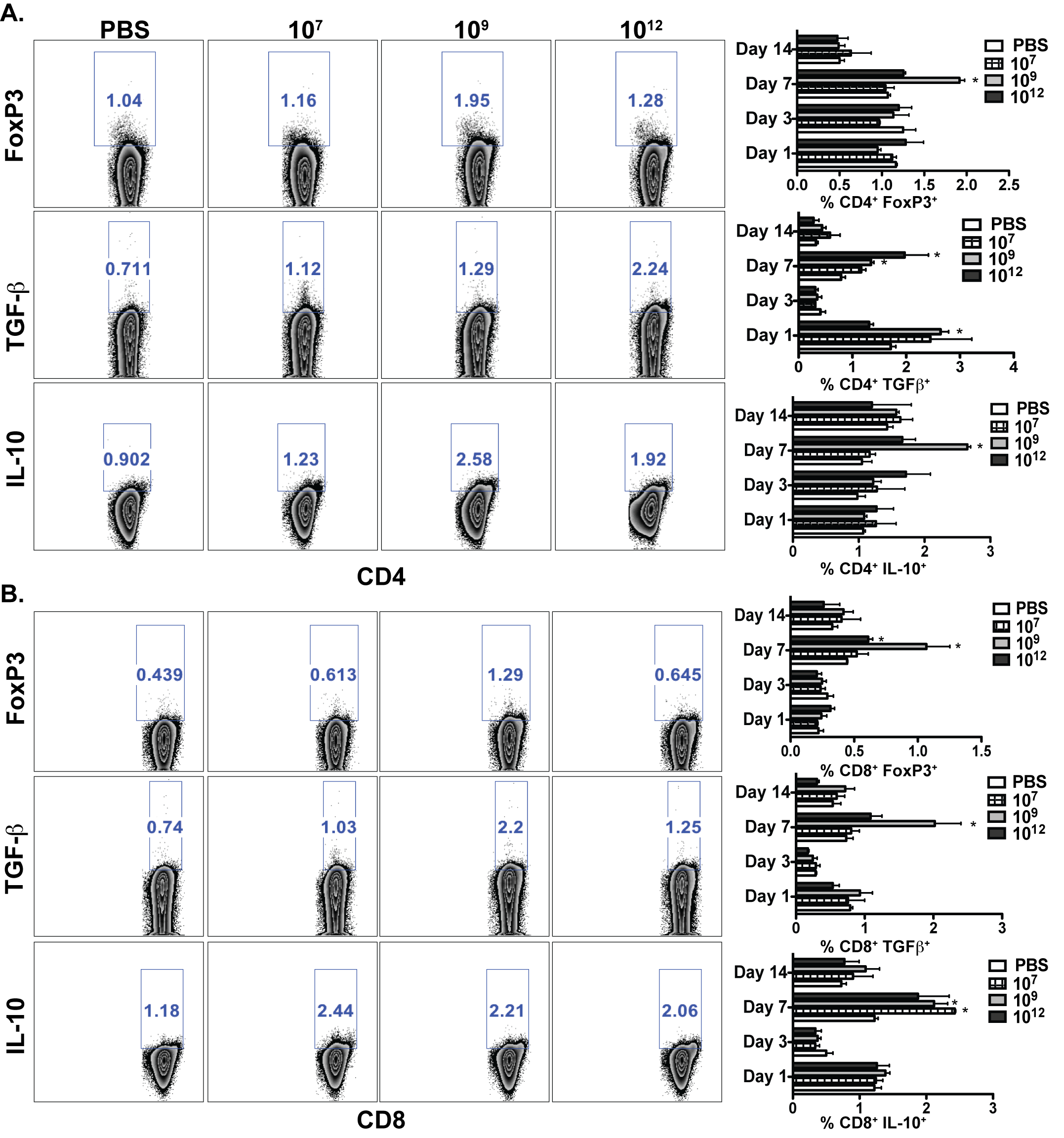

Supplement: Figure S4 — L. gasseri expressing PA-DCpep induce regulatory T cells in MLN. (A & B) C57BL/6 mice were orally gavaged with increasing doses of L. gasseri expressing PA-DCpep (107, 109 and 1012 CFU) or PBS, and MLNs were harvested after days 1, 3, 7 and 14, stained with antibodies against CD4, CD8, FoxP3, TGFβ and IL-10, and analyzed by flow cytometry. Data are representative of two independent experiments. Error bars represent ±SEM. *P<0.05 and **P<0.01 compared with PBS. (TIF) [file pone.0055143.s004.tif]

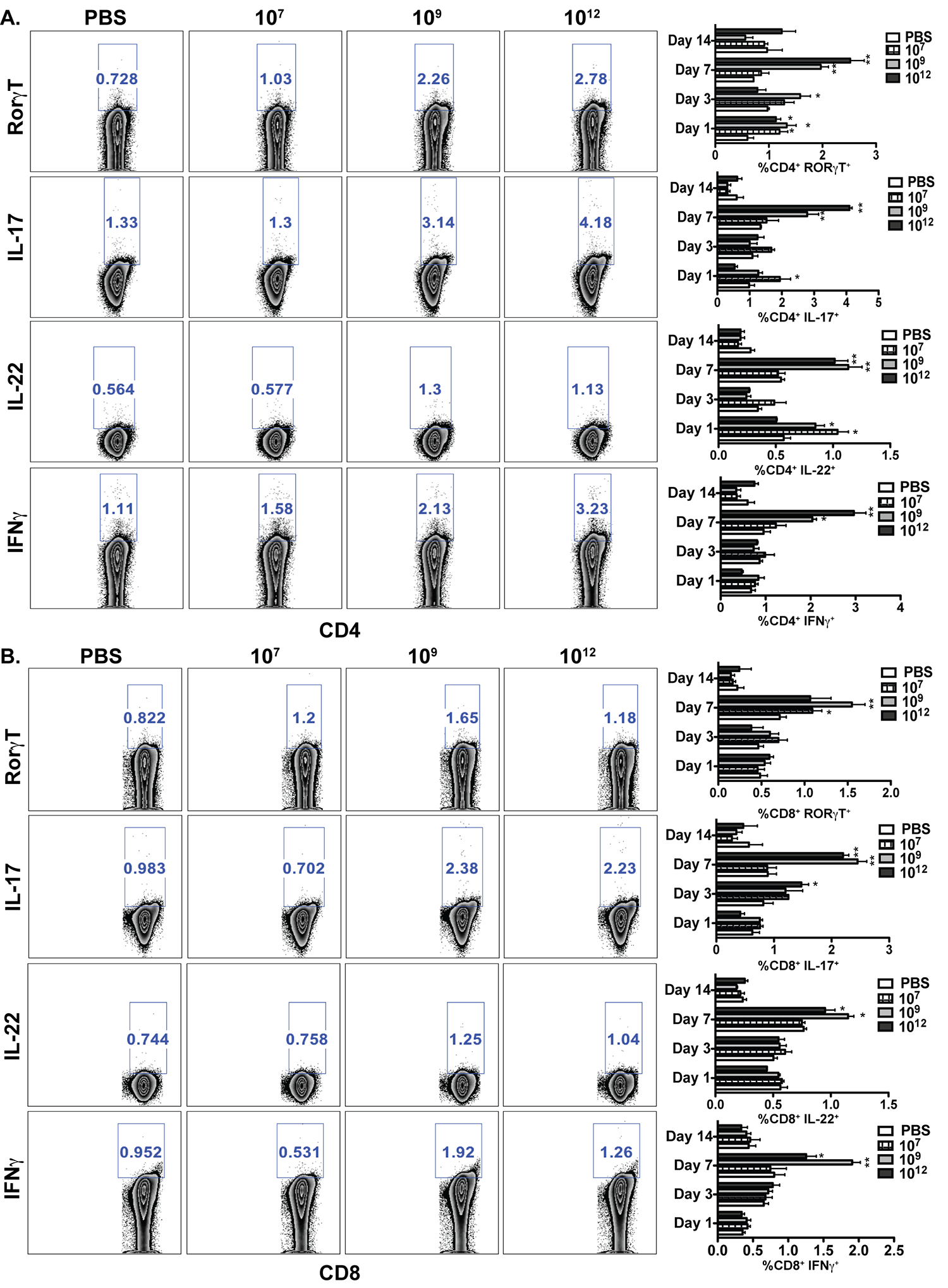

Supplement: Figure S5 — L. gasseri expressing PA-DCpep induce Th17 and Th1 cells in MLNs. (A & B) C57BL/6 mice were orally gavaged with increasing doses of L. gasseri expressing PA-DCpep (107, 109 and 1012 CFU) or PBS; MLNs were harvested after days 1, 3, 7 and 14, and stained with antibodies against CD4, CD8, RORγT, IL-17, IL-22 and IFNγ, and analyzed by flow cytometry. Data are representative of two independent experiments. Error bars represent ±SEM. *P<0.05 and **P<0.01 compared with PBS. (TIF) [file pone.0055143.s005.tif]

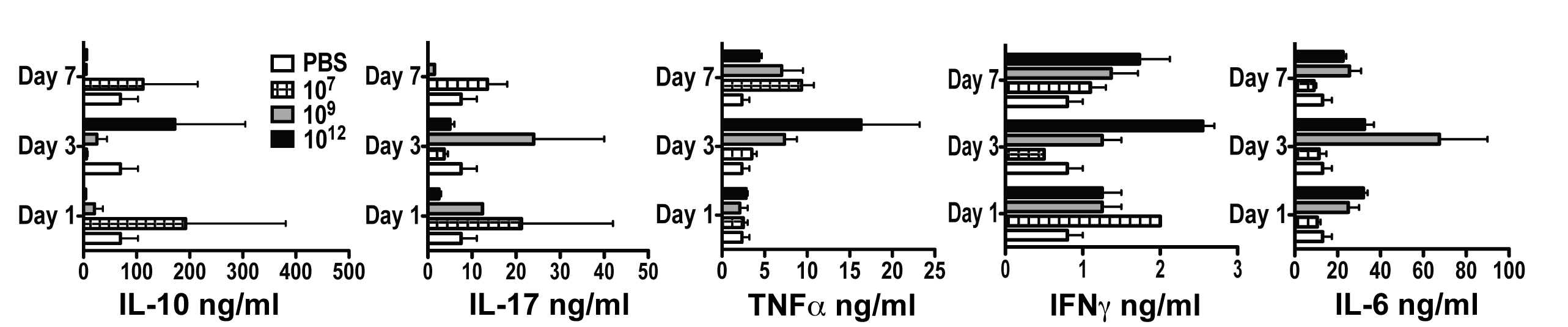

Supplement: Figure S6 — Augmentation of sera-cytokines by L. gasseri expressing PA-DCpep. C57BL/6 mice were orally gavaged with increasing doses of L. gasseri expressing PA-DCpep (107, 109 and 1012 CFU) or PBS; serum was collected after days 1, 3 and 7. ELISAs were performed to measure the secretion of cytokines. Data are representative of two independent experiments. (TIF) [file pone.0055143.s006.tif]
