# Supplementary material for: Colonic Immune Stimulation by Targeted Oral Vaccine
Source: PLoS One. 2013 Jan 30;8(1):e55143. doi: 10.1371/journal.pone.0055143 (PMC3559436; doi:10.1371/journal.pone.0055143)
Supplement: Table S1 — Primer Sequence used for qPCR. (DOCX) [file pone.0055143.s007.docx]

**Table S1. Primer Sequence used for qPCR**

| *tlr1* | 5’ CCGTGATGCACAGCTCCTTGGT 3’ |
| --- | --- |
|  | 5’AGGACGTTTCTGTAGGGGTGTCCA3’ |
| *tlr2* | 5’GGGGTGTGTGATGGCCGCTC3’ |
|  | 5’TGGAGGTTCGCACACGCTCG3’ |
| *tlr3* | 5’TGCTCAGGAGGGTGGCCCTT3’ |
|  | 5’CGGGGTTTGCGCGTTTCCAG3’ |
| *tlr4* | 5’AGGAGTGCCCCGCTTTCACCT3’ |
|  | 5’CCTTCCGGCTCTTGTGGAAGCC3’ |
| *tlr5* | 5’CGGTCCCGCCAGCCATTTCA3’ |
|  | 5’GGTGCGTGGGGGAACTCAGC3’ |
| *tlr6* | 5’ACCGTCAGTGCTGGAAATAGAGCTT3’ |
|  | 5’AGGCCAGGGCGCAAACAAAGT3’ |
| *tlr7* | 5’TCCCAGGCTGGAAGTTCTTGACC3’ |
|  | 5’CCTGGGGCCCATGACGGTCT3’ |
| *tlr8* | 5’GGCACAGAATTCTCCTCCATGCCC3’ |
|  | 5’AGACGGTGCGTTACCCCTGC3’ |
| *tlr9* | 5’ACGCAGCGCCCAAACTCTCC3’ |
|  | 5’GGCCAGGCAGCCAATCTCGG3’ |
|  |  |
| *nlrp1a* | 5’GGCCCCATAGAGGAGCAGGCA3’ |
|  | 5’TGCAAGCAGGAGACCAGTGGC3’ |
| *nod1* | 5’CTGAGCCGCGTGCCGGATAG3’ |
|  | 5’CACGCCTGTGCGAGCAGTGA3’ |
| *nod2* | 5’ATGAGCCGAGAGGACCGCGT3’ |
|  | 5’TTCCTCCCACGGGGCAACCA3’ |
| *nlrc3* | 5’AGGAGGGGAAGGCGCTCCAG3’ |
|  | 5’AGTGCCAGACCCTGGTGGCA3’ |
| *nlrc4* | 5’ACCACCACCACGGAGTGCCT3’ |
|  | 5’GGATTTGGGCCCACAGGCGT3’ |
| *nlrc5* | 5’TCCGCTGGAGGTGGGCTCAA3’ |
|  | 5’GGGCAGGGTCGGCAACTGTG3’ |
| *nlrp2* | 5’ACACACCTCAGCCTGGCCAAC3’ |
|  | 5’CAGGACCACAGCACCAGGGTC3’ |
| *nlrp3* | 5’GGCAAAAGGCTGTGCGGGGA3’ |
|  | 5’AAGGCTACCGGCCTCGTCGT3’3’ |
| *nlrp4e* | 5’TGCCTGATTTTCCCACAGAACCCA3’ |
|  | 5’AGCCCCAACTGCAAAATCTCCTGT3’ |
| *nlrp5* | 5’AGCAGGAGCAGACATCAGAAACCT3’ |
|  | 5’CGAACTTAGCAATCACATGGGCCT3’ |
| *nlrp12* | 5’GAAGAACGCCCCGAGCCACC3’ |
|  | 5’CCTCCCATCGGCCCAGTCCA3’ |
| *nlrp6* | 5’CCTGGACGGTGCAGACGAGC3’ |
|  | 5’CCTGTAGCGGCATGGCGTGT3’ |
| *nlrp10* | 5’AGGGGATGCGGCACCAGAGG3’ |
|  | 5’TGCCAAGCCCCGCTCGGTAG3’ |
| *nlrp9b* | 5’AGCATGTGTGCAGTTGTTGCTTGA3’ |
|  | 5’CCATAGCCAGATGAGCCCGCC3’ |
| *nlrp14* | 5’TCTCCACCGCTCTCCTGCACA3’ |
|  | 5’TGGCTGCTGGAGGGAGTGACA3’ |
| *asc* | 5’TGCCCATCGGCCTAAGCAGC3’ |
|  | 5’GGCAGCCGCGGTCACCTTTT3’ |
|  |  |
| *cd209b* | 5’TTGATGGTCAGCGGCAGCAGG3’ |
|  | 5’TCAGCAGGAGCCCAGCCAAGA3’ |
| *cd209d* | 5’TGCGCCAGGGACTGGACATT3’ |
|  | 5’ACCAGTTGGGCCCCCAGTTCC3’ |
| *clec4g* | 5’ACACACGTGGCCGGGGCTAT3’ |
|  | 5’CAGCCCCGAGTGCAGCATCAT3’ |
| *clec4b1* | 5’TTCATTGCGAGCTGTGTGGTCACT3’ |
|  | 5’TGGGCAACAGCTCCAGACTTTGT3’ |
| *clec4a2* | 5’CGCCCTGGTGATTCTATGCTGTGG3’ |
|  | 5’TCCTCCGTCAGAAGAGAGCCTTGT3’ |
| *clec4a4* | 5’GCACATGACACACTGGCCCACA3’ |
|  | 5’TGGGGAGTTGGCAAAAAGCTCCA3’ |
| *clec4n* | 5’GGTTTGCAGCATTGGCCCGC3’ |
|  | 5’GGTCCAGCAGACTCCCTTCCCT3’ |
| *clec4d* | 5’CGCTGGACGAGAGGAAGTGTGG3’ |
|  | 5’CCAGCTAACAGGACAGCAGGTCCA3’ |
| *clec4e* | 5’TGGGGGCTCACCTGGTGGTTA3’ |
|  | 5’GCCACTGACCCTCCACCACCT3’ |
| *cd207* | 5’GCTCCCGAAGCGCACTTCACA3’ |
|  | 5’GCAGGTGAAGGCCACGCAGA3’ |
| *clec9a* | 5’TGCTTCACCACTCCAAGCACCT3’ |
|  | 5’TCGTCACAACACACCATGCTCC3’ |
| *clec12b* | 5’GCGAGGCGCTGCCTTGAGTT3’ |
|  | 5’TGTCCTGCTGTGGATGGATGCTT3’ |
| *clec1a* | 5’ACCGCCCAGCCAGAAACACG3’ |
|  | 5’AGGCCGCCTGTCTACCCTCA3’ |
| *clec7a* | 5’TGGCCATCACCCAAACGTCGTG3’ |
|  | 5’TGCTTCCTGAAAGGGCAGCACC3’ |
| *clec10a* | 5’GCCCCAGAGGCCTCGTGTTTG3’ |
|  | 5’GGGAAGGAGCTGCTTTACCAGGC3’ |
| *mgl2* | 5’GGAACTGCAGGCAGGCCGAG3’ |
|  | 5’CAGCAGGCCACTTCCGAGCC3’ |
| *clec5a* | 5’TGGCACATGATCATCTCGGGGCT3’ |
|  | 5’GCTCTCCGTGGGGACGAAGC3’ |
| *klra17* | 5’CAACTTTTCCCCCATAACTGCAGGA3’ |
|  | 5’TGTGGCCAGCTTCTCTTGACCCT3’ |
| *clec12a* | 5’GCCATGCTGCAAAGCACAGCC3’ |
|  | 5’GCATTCCGAGCAGAGCAGGC3’ |
| *clec1b* | 5’CCCACACTGGCAGGCACTGA3’ |
|  | 5’AGCCATCACACGCCACCAAGA3’ |
